# Supplementary material for: Characterization of Three New Outer Membrane Adhesion Proteins in Fusobacterium necrophorum
Source: Microorganisms. 2023 Dec 12;11(12):2968. doi: 10.3390/microorganisms11122968 (PMC10745669; doi:10.3390/microorganisms11122968)
Supplement: Supplementary file 1 [file microorganisms-11-02968-s001.zip › microorganisms-2665932-supplementary.pdf]

Supplementary Figure

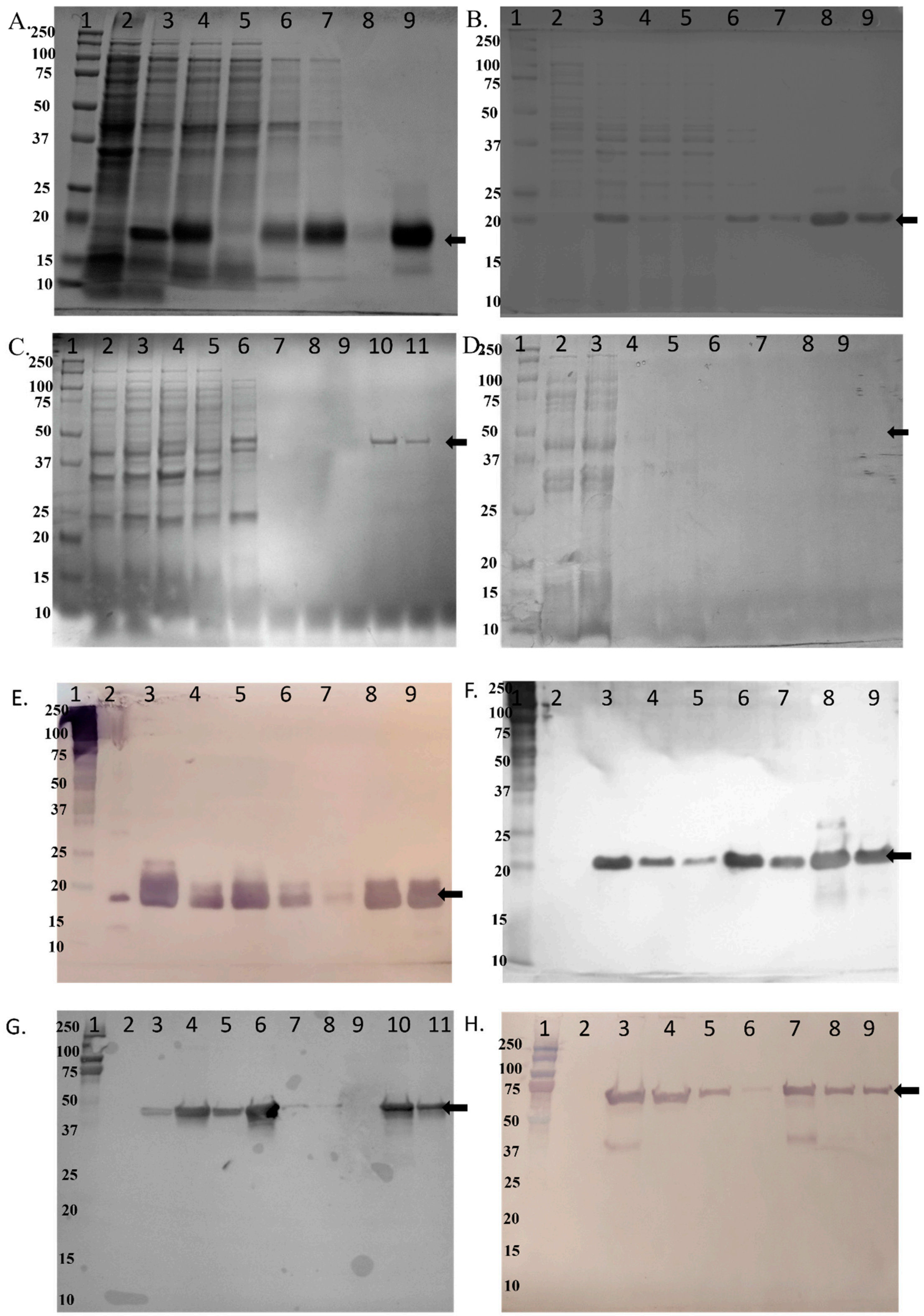

The supplemental figure displays SDS-PAGE (A-D) and western blot (E-H) analyses of aliquots obtained during various steps of IMAC-based recombinant protein purifications. The purified recombinant proteins, A, E. 17.5 kDa; B, F. 22.7 kDa; C, G. 43 kDa; and D, H. 66.3 kDa Outer Membrane Proteins (OMPs), are indicated by arrows. In each set, Lane 1- molecular marker; Lane 2- IPTG uninduced recombinant cell lysate; Lane 3 - IPTG induced recombinant cell lysate; and Lane 4- flowthrough of the protein-expressing bacterial lysate supernatant. The wash steps vary. Lane 5-7 for r17.5 kDa and r22.7 kDa, Lane 5-9 for r43 kDa, and Lane 5-8 for r66.3 kDa OMP. Subsequent lanes after washing steps correspond to the purified recombinant proteins: Lane 9 for r17.5kDa, Lanes 8 and 9 for r22.7 kDa, Lanes 10 and 11 for r43 kDa, and Lane 9 for 66.3 kDa OMPs.
